# Structural, surface morphological, optical and electrical properties of InxSey thin films, an absorber layer for photovoltaic cells fabricated by M-CBD method using different variables

**DOI:** 10.3906/kim-2104-7

**Published:** 2021-06-22

**Authors:** Fatih ÜNAL, Serkan DEMİR, Hasan MAMMADOV

**Affiliations:** 1 Giresun University, Central Research Laboratory, Application and Research Center, Giresun, Turkey; 2 Department of Industrial Engineering, Faculty of Engineering, Giresun University, Giresun, Turkey; 3 Department of Physics, Faculty of Arts and Sciences, Kafkas University, Kars, Turkey

**Keywords:** Thin films, InSe, J-V measurements, M-CBD, XRD, AFM

## Abstract

Mixed-phased In_x_Se_y_ thin film containing InSe, In_2_Se_3_ and In_6_Se_7_ phases was prepared by M-CBD method and characterized by X-ray diffraction, AFM, optical spectroscopy and J-V measurements. Structural, optical and electrical conductance properties were modified by annealing the films at different temperatures. Optical and morphological properties were also investigated dependently on temperature and concentration of cationic precursor solution. It has been observed with annealing that, the compositions of the phases changed, particle sizes increased, energy band gaps decreased and electrical conductivity increased. The photoconductivity of thin film was revealed by J-V measurements and slightly increased by annealing. From temperature-dependent J-V measurements, activation energies (E_a_) were calculated in low and high temperature regions and, found to be 0.03 eV for low temperature region and 0.8 eV for high temperature one.

## 1. Introduction

Indium selenide (In_x_Se_y_) thin films that can be constructed with various atomic combinations and phases such as InSe, In_2_Se_3_, In_4_Se_3_, In_9_Se_11_, In_6_Se_7,_ etc. have been verified as intriguing inorganic semiconductors during past few decades [1–6]. They attract particular interest of researchers owing to demonstrate noteworthy optical and photophysical properties that are promising for solar cells, photovoltaics, capacitors, microbatteries, field-effect transistors, strain engineering, nonlinear optics and miscellaneous nanoelectronic applications [7–13]. Not only their inherent polymorphism gives opportunity to be utilized as different layers for different purposes depending on the change of extent of band gap but also their junction with diverse substrates and layers enables the fabrication of different devices [1,2,5,14–18]. Especially, the absence or low density of dangling bonds at Van der Waals surfaces of In_x_Se_y_ make them peculiar for heterojunction processing [1,6,19,20]. However, coexistence of different phases in one layer and, presence of unproportional stoichiometries arising from loss of selenium during preparation can be major drawback of In_x_Se_y_ in order to achieve stoichiometric final product [4,20,21]. In many of techniques which were developed for efficient growing of semiconducting thin films on substrates, modified chemical bath deposition method (M-CBD) as modified version of chemical bath deposition (CBD) method is still one of the most appropriate techniques for In_x_Se_y_ growth to alleviate aforementioned handicaps [17,22–24]. It is relatively cheap, easy to apply and straightforward method aside from its advantages such as avoiding waste of materials adjusting rinsing time requiring none of restriction for substrate material, not inducing local overheat, etc. [17,23,24]. Therefore, we rationally benefited from M-CBD method in our study to grow In_x_Se_y_ phase on glass substrate. Optical, photoelectric, compositional and morphological features of the films were analyzed by XRD, AFM, UV-Vis and I–V characterization measurements. Thereby it is aimed to report an alternative for InSe based electronic devices (diode, photodetector, etc.) by varying molarity and annealing temperature parameters.

## 2. Methods

InSe thin films were deposited at room temperature on glass substrate of 30 × 26 × 2 mm dimensions by M-CBD method. The glass substrates were first boiled in chromic acid, then washed with hydrochloric acid followed by detergent water, and finally cleaned with acetone and distilled water. For cationic solutions, acidic (pH≈3) In_2_(SO_4_)_3_ solutions of different molar concentrations were used while for anionic ones, basic (pH≈12) Na_2_SeSO_3_ solution of 0.05 M was used. Optical properties were investigated using different molar concentrations of cationic precursor solutions while other measurements were made by using 0.07 M cationic precursor solution.

**Figure 1:**
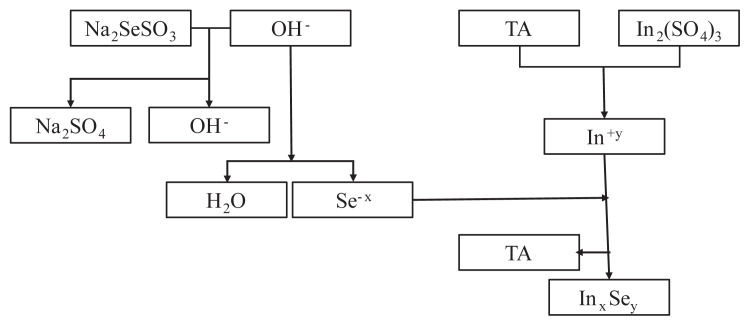
The growing scheme of In_x_Se_y_ thin films (TA: tartaric acid).

The substrates were 75 times immersed in In_2_(SO_4_)_3_ precursor solution for 30 s, in distilled water for 70 s, in Na_2_SeSO_3_ precursor solution for 10 s and distilled water for 70 s respectively, (Figures 1 [17] and 2). The two of the fabricated films were also annealed at 100 and 150 °C temperatures in air atmosphere for 1 h.

Structural analyses of thin films were performed with Rigaku D/Max-2200 XRD device in Ankara General Directorate of Mineral Research and Exploration (Cu-Kα radiation, λ = 1.5418 Å, 2θ = 10–70°). Surface morphologies of the films were probed by PSIAXE-100E model Atomic Force Microscopy (AFM). The thickness of the films were calculated using the equation t= m/ρ_s_A where t is thickness, m is the mass deposited on substrate, ρ_s_ is density and A is surface area of deposited film. Optical properties were investigated by Perkin-Elmer Lambda 25 UV-Vis spectrophotometer. The I–V measurements were carried out with Keithley 6486 pico-amperemeter and Pasco Scientific SF–9585. A power source using two probe technique in which silver metal was used for contacts.

## 3. Results and discussion

### 3.1. Structural properties

#### 3.1.1. XRD measurements

**Figure 3:**
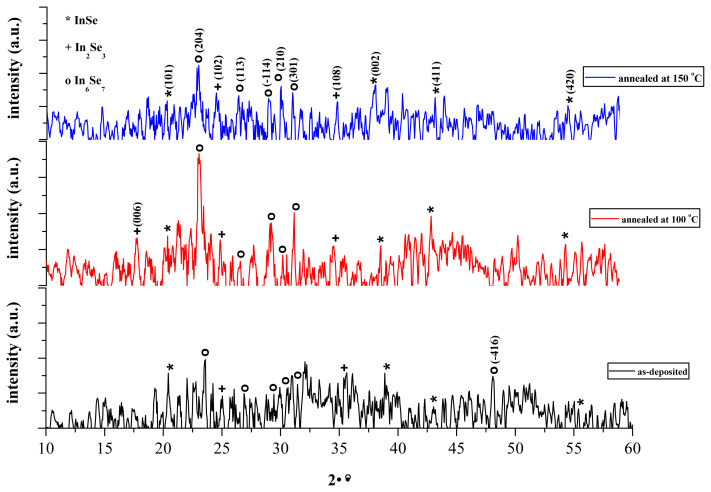
XRD spectra of the as-deposited and annealed thin films obtained from 0.07 M cationic precursor solution.

The thickness of thin film growth on glass substrate in 75 steps was estimated to be 112 nm. Lattice parameters and percentage compositions of phases are given in Table 1. The XRD spectra of thin films were depicted in Figure 3.

Polycrystal composition of In_x_Se_y_ film is understood from the presence of InSe, In_2_Se_3_ and In_6_Se_7_ phases in accordance with the previous literature [17] and the ratios of these phases change with annealing. The sharpest peaks for InSe(002), In_2_Se_3_ (108) and In_6_Se_7_(204) phases are observed at 2θ≈39°, 2θ≈35° and 2θ≈23°, respectively. The particle size (D), dislocation density (δ) and microstrain (ɛ) were calculated in these planes.

D can be calculated according to following Scherrer equation. Some important structural data are given in Table 2.[Fig f2-turkjchem-45-6-1761]


(1) $$D=\frac{0.90\cdot \lambda }{\beta \cdot cos\theta }$$

where D is particle size, λ is the wavelength of X-rays (1.5406 Å for CuKα), β is the full width at half maximum and θ is Bragg angle.

δ is calculated from Williamson and Smallman’s formula


(2) $$\delta =\frac{1}{D^{2}}lines/m^{2}$$

ɛ is obtained using the relation


(3) $$ɛ=\frac{\beta \cdot cos\theta }{4}$$

Generally, with annealing, particle size increases while dislocation density and lattice strain decreases (Table 2). The low dislocation density and lattice strain, and high particle size mean that the fine crystallization of film [25, 26].

#### 3.1.2. Surface morphology analyses

**Figure 4:**
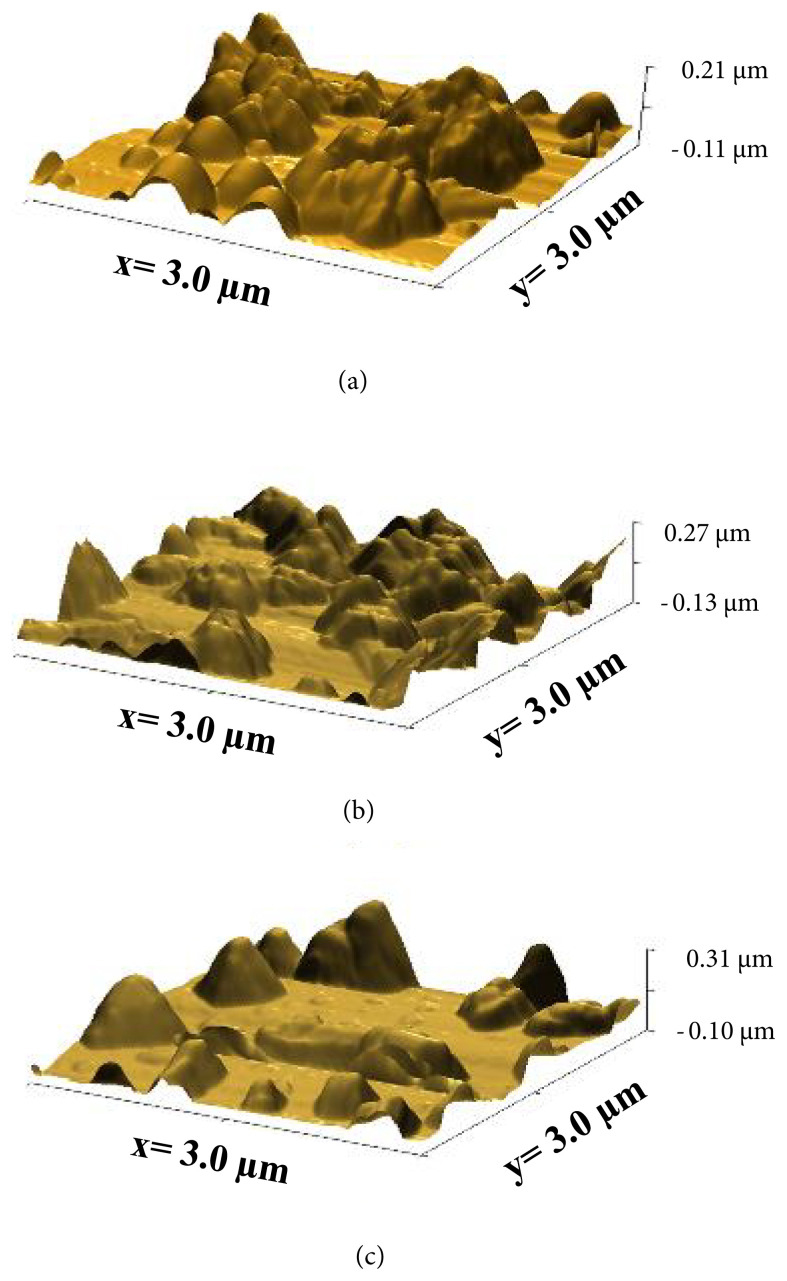
AFM images of thin films (a: as-deposited, b: 100 °C-annealed, c: 150 °C-annealed).

From AFM analyses, although clusters took place in some areas, it is observed that the film was growth on glass substrate in homogenous form in which none of cracks formed. Figure 4 represents AFM images of as-deposited, 100 °C-annealed and 150 °C-annealed thin films prepared from 0.07 M cationic precursor solutions. The particle sizes of thin films as-deposited, 100 °C-annealed and 150 °C-annealed were calculated to be 49.2 nm, 69.7 nm, and 106.8 nm, respectively. It is observed that the dimensions of thin films increased with the increase of annealing temperature as well as it is confirmed from XRD analyses. The growing of particle dimensions arises from the accumulation of smaller particles with thermal effect to form bigger ones. This situation is consistent with literature [20,27]. The increase of particle size also gives rise to noticeable increase of peak intensities, which means the increase of crystallization.

### 3.2. Optical properties

The optical absorption spectroscopy is one of the most used techniques to assign the energy band gap. The relation between absorption coefficient and energy band gap (E_g_) is given [28]


(4) $$\alpha (hv)\approx {\left(hv-E_{g}\right)}^{n}$$

where a is the absorption coefficient, *h_v_* is energy of photon and *E_g_* is energy band gap. The value of n is ½ for direct allowed transitions for InSe [29] materials. This technique plots ${\left(\alpha hv\right)}^{\frac{1}{n}}$ versus hυ. *E_g_* is assigned by extrapolating the straight line portion of the plot to the energy axis. The intercept gives the value of energy band gap.

The relation between absorbance (A) and transmittance (T) is given by


(5) A=-logT

and the reflection (R) is given by


(6) R=1-(A+T)

Then the refractive index (n) is given as [30, 31]


(7) $$n=\frac{1+\sqrt{R}}{1-\sqrt{R}}$$

**Figure 5:**
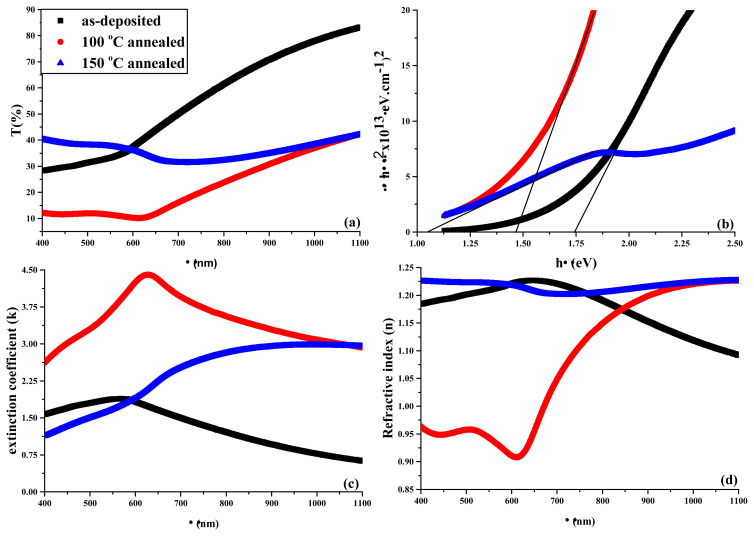
(a) Transmittances, (b) band gaps, (c) extinction coefficients, and (d) refractive indexes of the films obtained from 0.03 M precursor cationic solutions.

**Figure 6:**
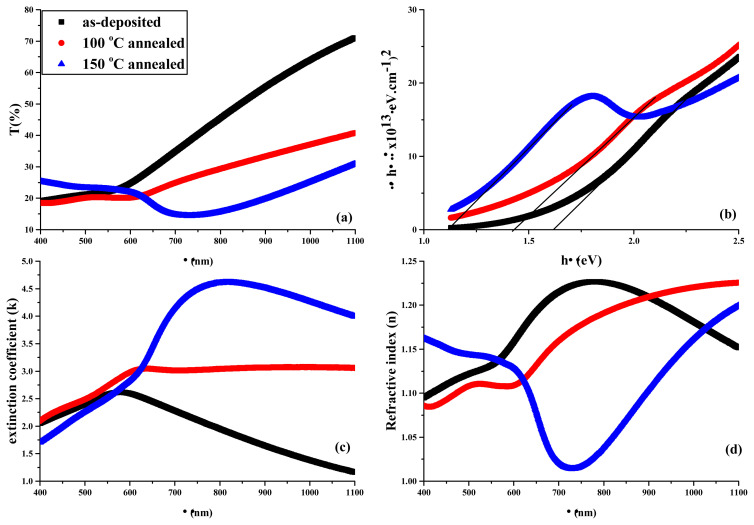
(a) Transmittances, (b) band gaps, (c) extinction coefficients, and (d) refractive indexes of the films obtained from 0.05 M precursor cationic solution.

**Figure 7:**
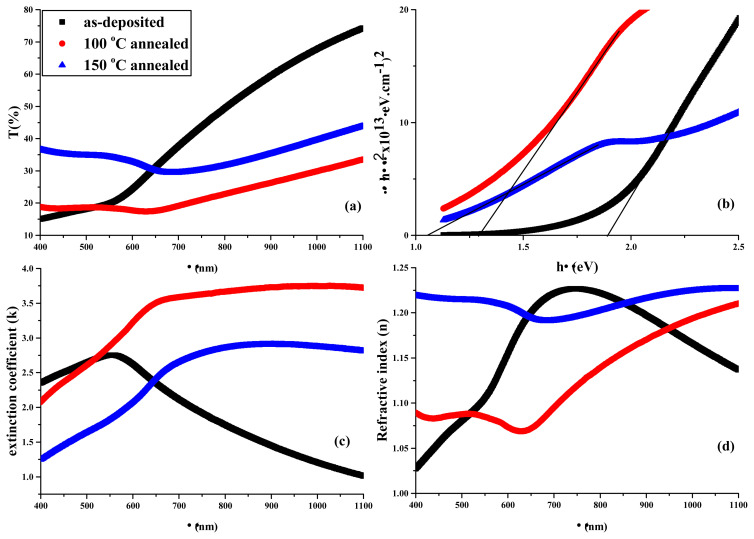
(a) Transmittances, (b) band gaps, (c) extinction coefficients, and (d) refractive indexes of the films obtained from 0.07 M precursor cationic solutions.

The relation between absorption coefficient (α) and extinction coefficient (k) is given by


(8) $$k=\frac{\lambda \cdot \alpha }{4\pi }$$

where α is calculated from the formula


(9) $$\alpha =\frac{2.303\cdot A}{d}$$

where d is thickness. The plots of T(%) vs. λ, band gap vs. hv, k and n vs. λ of as-deposited and annealed thin films prepared from various molar concentrations of cationic precursor solutions were measured and depicted in Figures 5a–5d, 6a–6d, and 7a–7d, respectively.

For visible region limit values, transmittance parameters were given in Table 3 while k and n values were given in Table 4.

In transmittance spectra of thin films, the highest transmittances were observed for as-deposited thin film among all thin films from 1100 to 600 nm while the lowest ones observed for 100 °C-annealed, 150 °C-annealed and 100 °C-annealed thin films from 0.03 M, 0.05 M and 0.07 M cationic precursor solutions, respectively. Within 400–600 nm range 150 °C-annealed thin films exhibit the highest transmittance while 100 °C-annealed thin films exhibit the lowest one among all thin films. Although reasonably low thickness of thin films, the low transmittance of fabricated thin films within visible region boundaries indicate that the fabricated thin films are convenient for devices running in visible region. The changes in molarities of cationic precursor solutions and annealing did not give rise to any trend in k and n values while the change in wavelengths give rise to trend in these values. For all cationic precursor solutions, k values in as-deposited films increased from 1100 nm to around 600 nm and decreased from around 600 nm to 400 nm. In 100 °C-annealed films, for 0.03 M precursor solution, k value increased from 1100 nm to around 640 nm and sharply decreased from around 640 nm to 400 nm while for 0.05 and 0.07 M precursor solutions, k values were almost constant from 1100 nm to 640 nm and sharply decreased again from 640 nm to 400 nm. In 150 °C-annealed films, for all solutions, k values were constant from 1100 nm to around 700 nm and sharply decreased from around to 700 nm to 400 nm. In case of n values, in as-deposited film, for 0.03 M precursor solution, n values increased from 1100 nm to 650 nm and decreased from 650 nm to 400 nm. For 0.05 M and 0.07 M precursor solutions, n values increased from 1100 nm to 750 nm and decreased from 750 nm to 400 nm. In 100 °C-annealed films, for 0.03 M precursor solution, n values sharply decreased from 1100 nm to 620 nm and increased from 620 nm to 400 nm. For 0.05 M precursor solution, n values decreased from 1100 nm to 600 nm, slightly increased from 600 nm to 500 nm, and decreased again from 500 nm to 400 nm. For 0.07 M precursor solution, n values decreased from 1100 nm to 630 nm, increased from 630 nm to 500 nm and stayed constant from 500 nm to 400 nm. In 150 °C-annealed films, for 0.03 M and 0.07 M precursor solutions, not a significant change was observed from 1100 nm to 400 nm. For 0.05 M precursor solution, n values sharply decreased from 1100 nm to 730 nm, increased from 730 nm to 600 nm and almost stayed constant from 600 nm to 400 nm. Trends similar to aforementioned were nearly observed in the study reported by Hossain et al. [32].

Table 5 lists the E_g_ values of the films. The increase of the concentrations of cationic precursor solution inconsistently influences E_g_ values in as-deposited thin film. However, it is observed that E_g_ is the function of concentration in 100 °C-annealed thin film while it is not reasonably influenced by concentration in 150 °C-annealed film. The reason for these changes probably arises from the change of In:Se stoichiometries. On the other hand, E_g_ values decreased with annealing. Besides, this can be attributed to several factors: i) particle growth effect, ii) quantum confinement effect, iii) the decrease of dispersions at particle boundaries, iv) the possibility of increase of structural defects, v) phase transitions with annealing treatment [33–35]. The first three of these reasons are also associated with each other.

The quantum confinement effect originating from systematic changes in energy levels as a function of internal dimension influences the particles of around 5 nm or lower sized [33]. As particle size increasing, quantum phenomena become slighter and separations in energy levels become narrower. Consequently, this causes a decrease of E_g_. Structural defects cause allowed states at band boundaries in forbidden area and hence decrease E_g_.

### 3.2. Electrical properties

Owing to the lowest transmittance values obtained for 100 °C-annealed film, only current density vs. voltage (J-V) characteristics of 100 °C-annealed thin film were compared with that of as-deposited one. J-V characteristics were investigated in dark and illuminated conditions of 100 W/m^2^ obtained from yellow lamb.

High activation energy values in semiconductors originate from trap states below conduction band or from electronic transitions between valance and conduction bands [36]. Low activation energy values are associated with hopping mechanism of electrons. This mechanism can be explained by weak interactions among donor and acceptor atoms. Impurity scatterings are effective in low temperature region while thermal scatterings are effective in high temperature region [37,38].

Also activation energies for as-deposited film were calculated within 298–428 K temperature range and corresponding logσ-10^3^/T plots were recorded. The calculations were made according to following equation [39, 40]:


(10) $$\sigma =\sigma_{0}\;\text{exp}(-\Delta E/kT)$$

**Figure 8:**
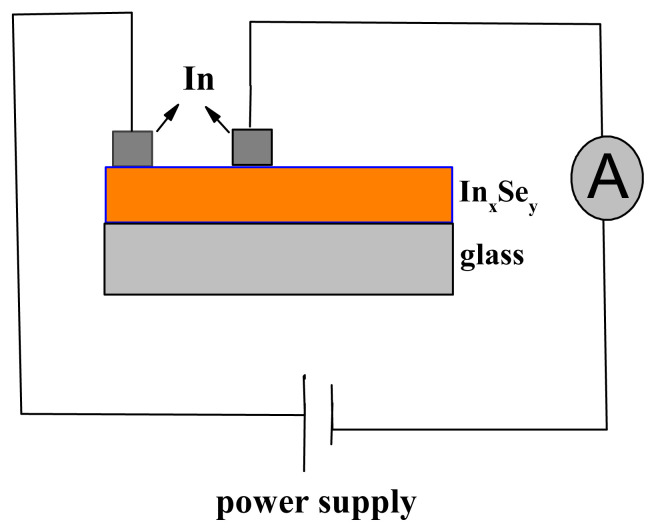
Schematic representation of the fabricated device.

**Figure 9:**
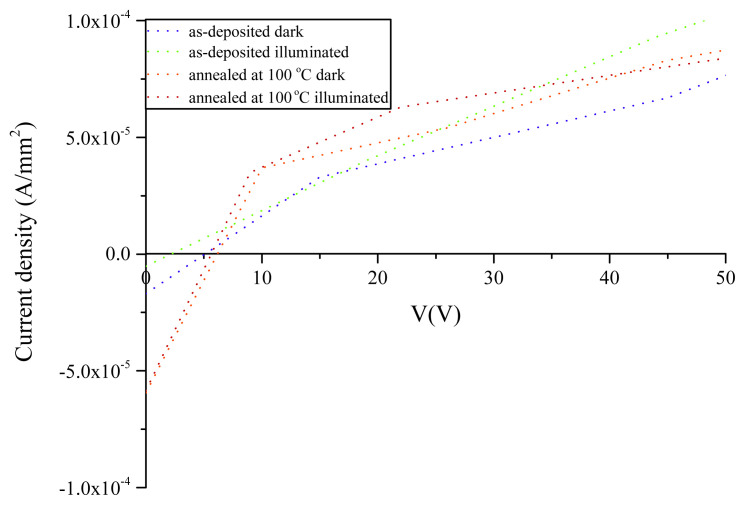
J-V characteristics of Ag/ In_x_Se_y_ /Ag and Ag/ In_x_Se_y_ (annealed)/Ag devices.

The schematized representation of fabricated device together with Ag parallel contacts is given in Figure 8. The J-V plots of Ag/ In_x_Se_y_ /Ag and Ag/ In_x_Se_y_ (annealed)/Ag devices were given in Figure 9.

Specific resistance values of as-deposited and annealed thin films are calculated as 1.8 × 10^8^ (ohm-cm) and 1.6 × 10^8^ (ohm-cm), respectively. As inferred from Figure 9, both as-deposited and annealed thin films are sensitive to light. The electrical conductance increases with annealing on account of the increase of particle size, the decrease of energy band gap and the change of distribution of the phases.

**Figure 10:**
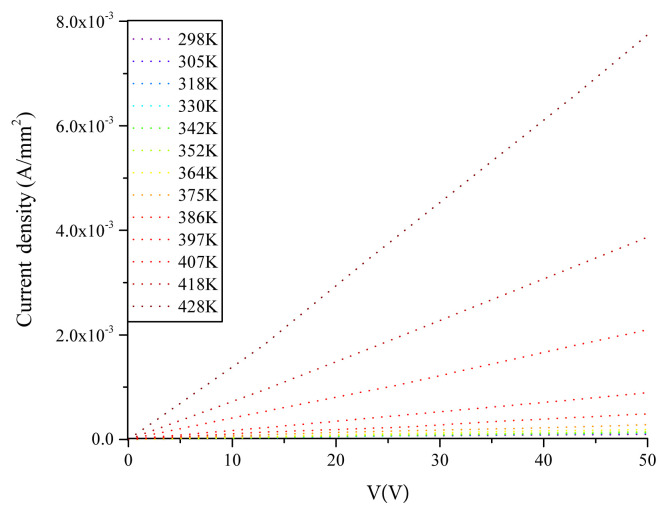
Temperature-dependent J-V characteristics of the as-deposited film.

**Figure 11:**
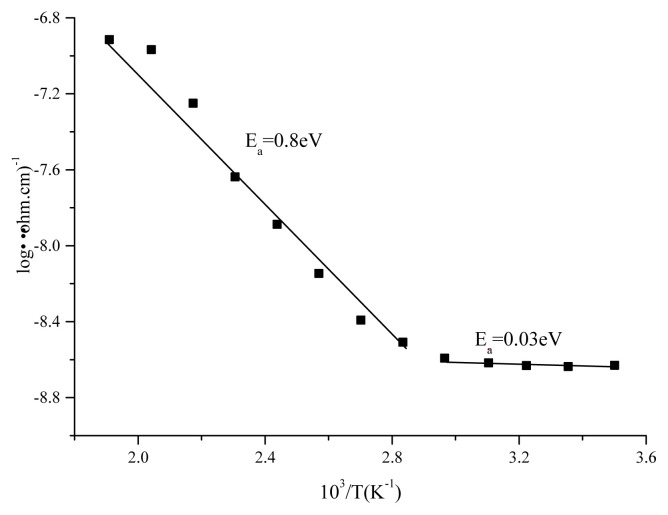
Arrhenius plot of the conductance of thin film (T = absolute temperature).

**Figure 12:**
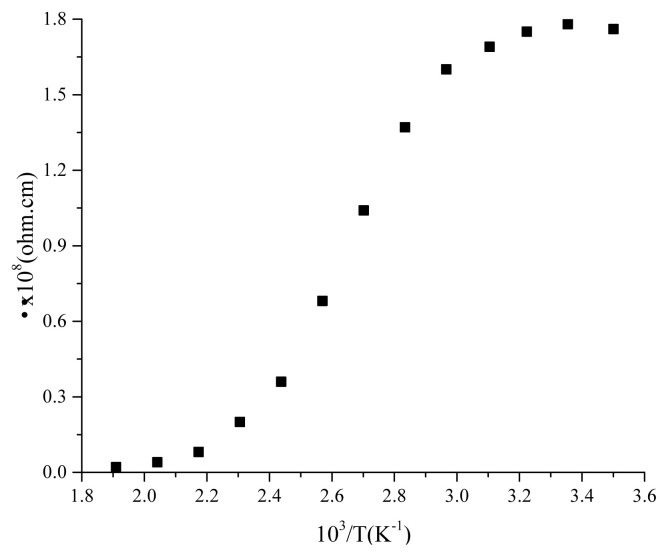
The plots of specific resistance versus 10^3^/T.

With temperature, the conductance of the device increases depending on the decrease of resistance according to J-V plot recorded within 298–428K range in Figure 10. This is a typical behaviour of semiconductor. The logσ-10^3^/T curve depicted in Figure 11 in this temperature range demonstrates that the activation energy in low temperature region in which doped conductivity is in question is 0.03 eV while the activation energy in high temperature region in which nondoped conductivity is in question is 0.8 eV. The increment in conductance is nonlinear owing to presence of different phases, amorphous structure of the film and formation of clusters on the surface as depicted from AFM images of the film [17]. In Figure 12, temperature dependence of specific resistance is also given. As expected, the specific resistance decreases with increasing temperature.

The fill factors (FF) of both as-deposited and annealed devices were calculated to be according to following equation;


(11) $$FF=\frac{J_{mpp}\cdot V_{mpp}}{J_{sc}\cdot V_{oc}}$$

where V_oc_ is open circuit voltage, J_sc_ short circuit current, J_mmp_ and V_mmp_ are the current density and voltage at the maximum power point (mmp), respectively.

FF values of the device from as-deposited film in dark and illumination are 0.15 and 0.25 respectively while these of the device from 100 °C-annealed film in dark and illumination are 0.13 and 0.24, respectively.

## 4. Conclusion

Using M-CBD method as one of the simple, straightforward, cheap and environmental-friendly, In_x_Se_y_ thin films were successfully prepared on the glass substrate. It is revealed that In_x_Se_y_ bears three different phases ( InSe, In_2_Se_3_, In_6_Se_7_). With annealing, the composition of the phases changed, particle sizes increased, energy band gaps decreased, electrical conductivities and photoconductivies increased. Concentration-dependence of optical properties using cationic precursor solution (0.03 M, 0.05 M, 0.07 M) was additionally investigated. The lowest transmittance, the lowest n and the highest k in visible region was observed for 0.05 M–150 °C-annealed film. From as-deposited to 100 °C-annealed film, the sharpest narrowing of energy band gap was detected for the film prepared from 0.07 M cationic solution. It is evident from temperature-dependent J-V plots that the film which incorporates mixed phases behave as classical semiconductor with reasonable specific resistance values of 1.8 × 10^8^ (ohm-cm) and 1.6 × 10^8^ (ohm-cm) for as-deposited and 100 °C-annealed films, respectively. Consequently, the lowest transmittance of the film placed around 600–750 nm demonstrates that the prepared film is potential candidate for visible-region photodetectors such as solar cells, photodiodes, etc. In further studies, corresponding properties can be developed by deposition with different semiconductors and heterojunctions.

Contribution of authors Fatih Ünal: Conceptualization, Investigation, Formal analysis, Writing – review and editing. Serkan Demir: Formal analysis, Writing – review and editing. Hasan Mammadov: Investigation, Funding acquisition.

## Figures and Tables

**Figure 2 f2-turkjchem-45-6-1761:**
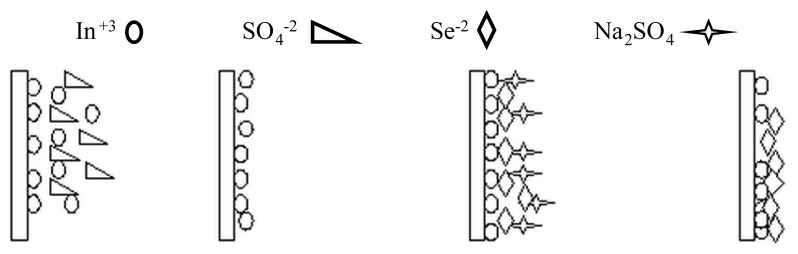
The formation of In_x_Se_y_ thin films by M-CBD method.

**Table 1:** Lattice parameters of the phases that constituting thin film.

Formula			
	InSe	In_2_Se_3_	In_6_Se_7_
**Temperature (K)**	300		
**Ref. No (COD)**	96-154-0842	96-152-8775	96-210-6209
**Wavelength (Å)**	1.5418 (Cu-Kα)		
**Crystal system**	Monoclinic	trigonal (hexagonal axes)	Monoclinic
**Space group**	P 1 2/m 1 (10)	R −3 m (166)	P 1 21 1(4)
**Unit cell dimensions (Å)**			
***a***	11.020	4.05	9.430
***b***	4.110	-	4.063
***c***	4.610	29.410	18.378
***B (°)***	87.200	-	109.340
**Cell volume (Å** **^3^** **)**	208.54	417.76	664.40
**Z**	6	6	2
	**Composition (%)**		
**As-deposited**	25.2	21.2	53.6
**100 °C-annealed**	28.9	37.9	33.1
**150 °C-annealed**	27.4	29.6	43.0

Lattice parameters and compositions were calculated using Match! program.

**Table 2:** Some important structural parameters of the films.

2θ	Condition	Phase	(hkl)	FWHM (β)	Particle size (D) (nm)	Lattice strain (ɛ)	Dislocation density (δ) × 1015
23.52703	As-deposited	In_6_Se_7_	204	0.20655	37.66379905	0.050552979	0.705
35.54204		In_2_Se_3_	108	0.33524	22.5724337	0.079810913	1.963
38.51931		InSe	002	0.33308	22.52198203	0.078609665	1.971
23.39656	Annealed-100ºC	In_6_Se_7_	204	0.38039	20.45610342	0.09312222	2.389
35.06916		In_2_Se_3_	108	0.26774	28.3003165	0.063824885	1.248
39.14171		InSe	002	0.1634	45.82175503	0.038490012	0.476
23.2444	Annealed-150ºC	In_6_Se_7_	204	0.11952	65.12240399	0.029267378	0.236
35.35063		In_2_Se_3_	108	0.12022	62.97806785	0.028636175	0.252
39.11148		InSe	002	0.11533	64.92653007	0.027169335	0.237

**Table 3:** Transmittance parameters of thin films.

λ (nm)	Transmittance (T)		
	As-deposited	As-deposited	150 °C-annealed
**0.03 M**			
700	50.02649	50.02649	31.31843
400	28.22279	28.22279	40.2161
**0.05 M**			
700	34.87386	34.87386	14.75367
400	19.13375	19.13375	25.31629
**0.07 M**			
700	37.60971	37.60971	29.43744
400	15.03488	15.03488	36.57632

**Table 4:** Extinction coefficients and refractive indexes of the films.

λ (nm)	Extinction coefficients (k)			Refractive indexes (n)		
	As-deposited	100 °C-annealed	150 °C-annealed	As-deposited	100 °C-annealed	150 °C-annealed
**0.03 M**						
700	1.49764	3.9564	2.51921	1.22072	1.04767	1.20093
400	1.56829	2.61362	1.12926	1.18385	0.96362	1.22499
**0.05 M**						
700	2.27943	3.01226	4.15622	1.21484	1.15929	1.02099
400	2.05013	2.09153	1.70302	1.09475	1.08577	1.16244
**0.07 M**						
700	2.11458	3.58624	2.65259	1.22147	1.09493	1.19125
400	2.34901	2.07497	1.24687	1.02711	1.08938	1.21906

**Table 5:** Assigned energy band gap values.

	Energy band gaps (eV)		
	As-deposited	Annealed at 100 °C	Annealed at 150 °C
Glass/ In_x_Se_y_ 0.03 M C.S	1.73	1.59	1.12
Glass/ In_x_Se_y_ 0.05 M C.S	1.63	1.45	1.16
Glass/ In_x_Se_y_ 0.07 M C.S	1.87	1.3	1.12

C.S: cationic solution.
